# Study from the United States: increased prevalence of kidney stones in patients with high weight-adjusted waist index

**DOI:** 10.3389/fnut.2023.1171775

**Published:** 2024-01-17

**Authors:** Zhaohua Gui, Longshan Yu, Yan Chen, Mingxun Zhang, Jie He, Yunwu Hao

**Affiliations:** ^1^Division of Life Sciences and Medicine, Department of Pathology, The First Affiliated Hospital of USTC, University of Science and Technology of China, Hefei, Anhui, China; ^2^Division of Life Sciences and Medicine, Department of Emergency Medicine, The First Affiliated Hospital of USTC, University of Science and Technology of China, Hefei, Anhui, China; ^3^Department of General Practice, Wuhu City Second People’s Hospital, Wuhu, China; ^4^Department of Urology, Lu’an Hospital Affiliated of Anhui Medical University (Lu an City People’s Hospital), Lu’an, Anhui, China

**Keywords:** kidney stone prevalence, weight-adjusted waist index (WWI), NHANES, cross-sectional study, visceral obesity

## Abstract

**Objective:**

Using data from NHANES 2007–2018, to examine the association between WWI (weight-adjusted waist index) index and prevalence of kidney stones.

**Methods:**

Using multiple logistic regression analysis of the National Health and Nutrition Examination Survey (NHANES) 2007–2018, we evaluated the association between WWI index and the prevalence of kidney stones, followed by subgroup analysis of sensitive populations. Smooth curve fitting was used to determine whether there was a non-linear relationship between the WWI index and kidney stone prevalence, and threshold effect analysis was used to test this relationship.

**Results:**

Among 29,280 participants, 2,760 self-reported renal calculi. After adjustment for all confounders, there was a positive association between WWI and kidney stone prevalence (OR = 1.20, 95% CI: 1.12, 1.28), and this positive association was stronger with increasing WWI (and *P* = 0.01 for trend). Our results indicate a non-linear positive correlation between WWI index and kidney stones, with the saturation threshold effect analysis and the most important threshold value at 11.02. According to subgroup analysis, WWI showed the strongest association with kidney stone prevalence in participants aged 20–39 years, males, other US ethnic groups, and participants without hypertension and diabetes.

**Conclusion:**

Increased WWI is positively associated with increased incidence of kidney stones, and increased WWI is a high risk for kidney stones that should be treated with caution. This association should be more pronounced in people between the ages of 20 and 39 years, in men, in other US ethnic populations, and in participants who do not have hypertension or diabetes.

## 1 Introduction

Kidney stones are a common urinary disease, accounting for 40–50% of all urinary stone diseases, caused by the abnormal accumulation of crystalline substances in the kidney (1). The prevalence of kidney stones in developed countries is 5∼15%, the prevalence of kidney stones in China is gradually increasing, the prevalence of adults is 6.4%, and the age of onset is also younger (2). In addition, kidney stones are prone to recurrence after treatment, and about one-third of patients with a first attack will have stone recurrence (3), and a higher risk in patients with stone recurrence (4), putting a heavy burden on the health system (5). The estimated annual cost in 2005 was more than $5 billion (6). In addition, the longest kidney stone accompanied by hematuria and lower back pain, if not treated often lead to hydronephrosis, will seriously damage kidney function (7).

The pathogenesis of kidney stones is more complex, and there are many triggering factors, such as dietary, metabolic, pharmacological, and environmental factors (2). With the change in dietary structure and daily lifestyle, obesity has gradually become a major problem for human health. Obesity can not only lead to the accumulation of body fat, affect the body shape, but also cause visceral fat accumulation, affect the function of related organs. The analysis of the Health Professionals Follow-up Study and the Nurses’ Health Study I and II identified associations between obesity, diabetes, and kidney stone disease (8, 9). Currently, the most commonly used measure of obesity is BMI (Body Mass Index) (10). However, BMI has some limitations, such as the inability to distinguish between lean body mass and fat body weight (11). However, increased visceral fat in central obesity may be more likely to reflect adverse metabolic characteristics, which is receiving more attention from researchers (12). Some studies have also shown that visceral adiposity is more associated with diabetes, hypertension, and hyperuricemia than subcutaneous adiposity (13–15). Methods used to assess the content and distribution of body fat include: densitometry (DXA), MRI, CT, and mechanical methods that provide high accuracy in assessing body fat, with the first three methods providing an image of fat and its location in the body (16). However, these methods are technically complex procedures, and their cost and time requirements are too high for them to be used on a routine basis in clinical practice. The weight-adjusted waist circumference index (WWI) is a new anthropometric index of obesity proposed in 2018 (17). Compared with BMI, WWI can better distinguish between fat and muscle mass components, and mainly reflects the problem of central obesity, which is not affected by body weight (18). Previous studies have reported that increased WWI is associated with many diseases, such as hypertension, hyperuricemia (19–22). However, whether there is no association between WWI and the prevalence of kidney stones has not been reported. We hypothesized that WWI may be associated with the development of kidney stones. To address this question, while controlling for a variety of known risk factors for kidney stones, we examined the association between WWI and kidney stones in a nationally representative survey. This is the first study to show a possible association between WWI and renal calculi.

## 2 Materials and methods

### 2.1 Study population

**FIGURE 1:**
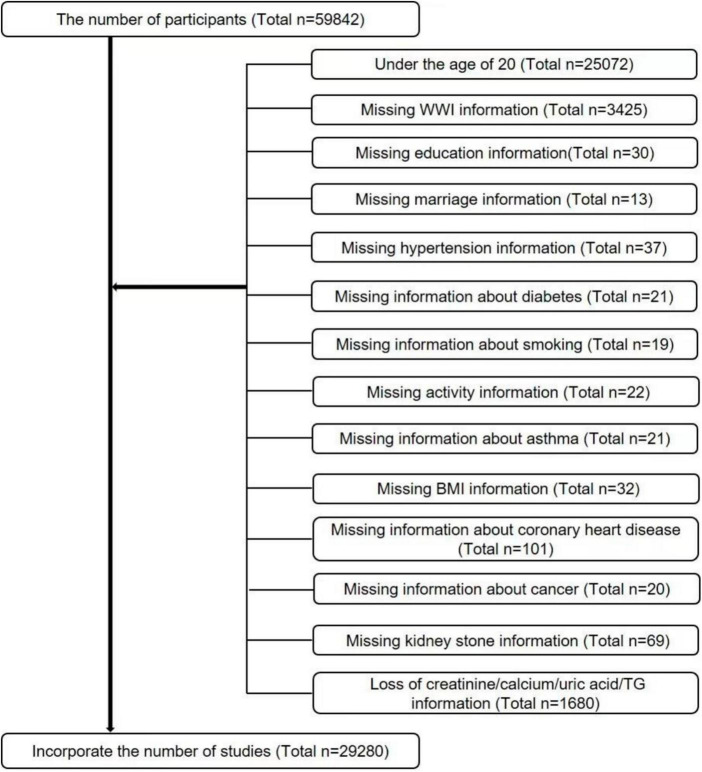
Flow chart for participants.

The data for this study were obtained by the Centers for Disease Control and Prevention (CDC) from the NHANES database, using big data mining techniques (23, 24). The NHANES is a cross-sectional survey that has been updated and new data have been released every 2 years since 1999. Since the questionnaire for kidney stones was only available after 2007, we selected data from six cycles from 2007 to 2018 based on our study. Because kidney stones were only investigated in people aged 20 years and older, we screened the study population based on the purpose of our study, and detailed inclusion and exclusion criteria are shown in Figure 1. In summary, a total of 29,280 cases were included in this study. Of these, 2,760 had a self-reported history of nephrolithiasis.

### 2.2 Data collection and definition

The WWI Index was designed to serve as an exposed variable. WWI was calculated for each participant as WC in centimeters divided by the square root of body weight in kilograms, then rounded to two decimal places. The primary outcome measure was whether the participant had urinary stones. Studies have validated the accuracy of self-reported stone status. If the participant answered “yes,” they had a kidney stone. The incidence of kidney stones was used as the outcome measure.

A multivariable adjusted model was used to summarize potential covariates that might confound the association between WWI index and kidney stones. The covariates in our study included sex, age (years), race, education level, poverty income ratio (PIR), marital status, alcohol consumption, physical activity, cholesterol level (mg/dl), serum glucose (mg/dl),serum calcium (mg/dl),serum triglyceride (mg/dl)s, serum creatinine (mg/dl), serum uric acid (mg/dl), smoking status, hypertension, diabetes, coronary heart disease, cancer. There are also dietary intake factors, including energy intake, fat intake, sugar intake, and water intake, and all participants are eligible for two 24-h dietary recalls, and our analysis will use the average consumption of the two recalls. To deal with missing values, we converted them to categorical variables. All of the detailed procedures used to measure the study variables are publicly available at the following website: www.cdc.gov/nchs/nhanes/.

### 2.3 Statistical methods

All statistical analyses were conducted according to CDC guidelines using appropriate NHANES sampling weights and accounting for complex multistage cluster surveys. The NHANES detailed weight analysis guide was provided on the official website, NHANES analysis guide, which constructed new sampling weights for the combined survey cycle by dividing the 2-year weight for each cycle by 6. The weights provided on the official website are resolved using the Survey Design R package in the R language. Continuous variables were assessed using survey-weighted linear regression, along with a survey-weighted chi-squared test (for categorical variables) to assess group differences. Continuous variables were expressed as weighted survey means and 95% CIs, and categorical variables were expressed as weighted survey means and 95% CIs. Following the STROBE guidelines (25), three multivariate regression models were constructed. Model 1 was unadjusted for covariates. Model 2 was adjusted for gender, age, race, education level, and marital status. Model 3 was adjusted for all variables, excluding collinearity (collinearity in VIF screening, considered collinearity if the VIF value is greater than 5). In the sensitivity analysis, WWI was converted from a continuous variable to a categorical variable (tertiles) to assess its robustness. The test for linear trend was performed using the tertiles of WWI as a continuous variable. We also used generalized additive models (GAM) and smooth curve fitting to account for non-linearity of WWI with albuminuria and each stratification. When a non-linear correlation was observed, a two-segment linear regression model (segmented regression model) was used to fit each interval and calculate the threshold effect. A *p* < 0.05 was considered statistically significant. All analyses were performed using Empower software (X&Y Solutions, Inc., Boston, MA, USA)^1^ and R version 4.0.2 (The R Foundation).^2^

## 3 Results

### 3.1 Participant characteristics

The baseline demographic characteristics of the included participants are shown in Table 1. WWI index of 11.25 (11.21, 11.30) in the stone group, higher than the normal group of 10.95 (10.92, 10.97), *P* < 0.001.

**TABLE 1:** Baselines characteristics of participants, weighted.

Characteristic	Non-stone formers (*n* = 26,520)	Stone formers (*n* = 2,760)	*P*-value
Age (years)	46.56 (46.10, 47.03)	53.15 (52.51, 53.78)	<0.0001
Serum cholesterol (mg/dl)	194.10 (193.10, 195.10)	192.36 (189.87, 194.85)	0.1493
Serum glucose (mg/dl)	98.72 (98.14, 99.31)	106.04 (104.37, 107.72)	<0.0001
BMI (kg/m^2^)	28.84 (28.67, 29.01)	30.50 (30.17, 30.83)	<0.0001
Serum triglycerides (mg/dl)	150.60 (147.98, 153.22)	172.92 (162.02, 183.82)	0.0001
Serum uric acid (mg/dl)	5.39 (5.36, 5.41)	5.59 (5.52, 5.65)	<0.0001
WWI index	10.95 (10.92, 10.97)	11.25 (11.21, 11.30)	<0.0001
Serum calcium (mg/dl)	9.39 (9.38, 9.41)	9.37 (9.34, 9.40)	0.0621
Serum creatinine (mg/dl)	0.87 (0.87, 0.88)	0.93 (0.91, 0.94)	<0.0001
Gender (%)			<0.0001
Male	47.75 (47.05, 48.45)	55.43 (52.74, 58.09)	
Female	52.25 (51.55, 52.95)	44.57 (41.91, 47.26)	
Race (%)			<0.0001
Mexican American	14.98 (13.09, 17.10)	11.32 (9.36, 13.63)	
White	65.66 (62.79, 68.43)	76.91 (73.78, 79.77)	
Black	11.15 (9.77, 12.69)	5.68 (4.69, 6.86)	
Other race	8.20 (7.36, 9.14)	6.09 (4.89, 7.56)	
Education level (%)			0.1076
Less than high school	20.41 (18.98, 21.91)	19.82 (17.89, 21.90)	
High school	28.65 (27.47, 29.86)	31.39 (28.58, 34.33)	
More than high school	50.94 (49.07, 52.81)	48.79 (45.64, 51.95)	
Marital status (%)			<0.0001
Cohabitation	63.38 (62.10, 64.65)	69.43 (66.85, 71.90)	
Solitude	36.62 (35.35, 37.90)	30.57 (28.10, 33.15)	
Alcohol (%)			0.7312
Yes	61.16 (59.69, 62.62)	60.03 (56.97, 63.02)	
No	18.49 (17.44, 19.60)	19.26 (17.06, 21.67)	
Unclear	20.34 (19.24, 21.49)	20.71 (18.02, 23.67)	
High blood pressure (%)			<0.0001
Yes	29.67 (28.67, 30.69)	46.26 (43.35, 49.19)	
No	70.33 (69.31, 71.33)	53.74 (50.81, 56.65)	
Diabetes (%)			<0.0001
Yes	8.50 (8.03, 9.01)	17.50 (15.76, 19.38)	
No	91.50 (90.99, 91.97)	82.50 (80.62, 84.24)	
Smoked (%)			<0.0001
Yes	43.59 (42.37, 44.82)	49.46 (46.60, 52.32)	
No	56.41 (55.18, 57.63)	50.54 (47.68, 53.40)	
Physical activity (%)			0.0059
Never	26.02 (25.04, 27.03)	29.73 (27.51, 32.06)	
Moderate	31.95 (30.97, 32.95)	31.39 (29.10, 33.78)	
Vigorous	42.03 (40.91, 43.15)	38.87 (36.15, 41.67)	
Asthma (%)			0.0041
Yes	14.49 (13.81, 15.20)	17.25 (15.43, 19.23)	
No	85.51 (84.80, 86.19)	82.75 (80.77, 84.57)	
Coronary artery disease (%)			<0.0001
Yes	3.07 (2.70, 3.49)	6.27 (5.19, 7.55)	
No	96.93 (96.51, 97.30)	93.73 (92.45, 94.81)	
Cancers (%)			<0.0001
Yes	9.33 (8.85, 9.83)	15.78 (14.24, 17.45)	
No	90.67 (90.17, 91.15)	84.22 (82.55, 85.76)	
PIR (%)			0.117
<1.3	20.13 (18.89, 21.43)	18.07 (16.32, 19.96)	
≥1.3<3.5	32.50 (31.25, 33.77)	35.03 (32.44, 37.70)	
≥3.5	40.04 (38.17, 41.94)	40.02 (36.73, 43.40)	
Unclear	7.34 (6.70, 8.03)	6.89 (5.63, 8.39)	
Total sugar (%)			0.9867
Lower	36.47 (35.61, 37.34)	36.70 (33.90, 39.59)	
Higher	37.21 (36.25, 38.18)	37.14 (34.21, 40.17)	
Unclear	26.32 (25.51, 27.15)	26.16 (23.86, 28.60)	
Total Kcal (%)			0.2319
Lower	39.07 (38.25, 39.90)	40.23 (38.01, 42.50)	
Higher	46.05 (45.06, 47.04)	46.47 (43.76, 49.20)	
Unclear	14.88 (14.08, 15.70)	13.30 (11.57, 15.24)	
Total water (%)			0.0413
Lower	38.86 (37.95, 39.79)	37.20 (34.68, 39.80)	
Higher	46.26 (45.32, 47.20)	49.50 (46.68, 52.32)	
Unclear	14.88 (14.08, 15.70)	13.30 (11.57, 15.24)	
Total fat (%)			0.0413
Lower	38.86 (37.95, 39.79)	37.20 (34.68, 39.80)	
Higher	46.26 (45.32, 47.20)	49.50 (46.68, 52.32)	
Unclear	14.88 (14.08, 15.70)	13.30 (11.57, 15.24)	

For continuous variables: survey-weighted mean (95% CI), *P*-value was by survey-weighted linear regression (svyglm).

For categorical variables: survey-weighted percentage (95% CI), *P*-value was by survey-weighted Chi-square test (svytable).

### 3.2 Kidney stone prevalence was associated with a higher WWI index

**FIGURE 2:**
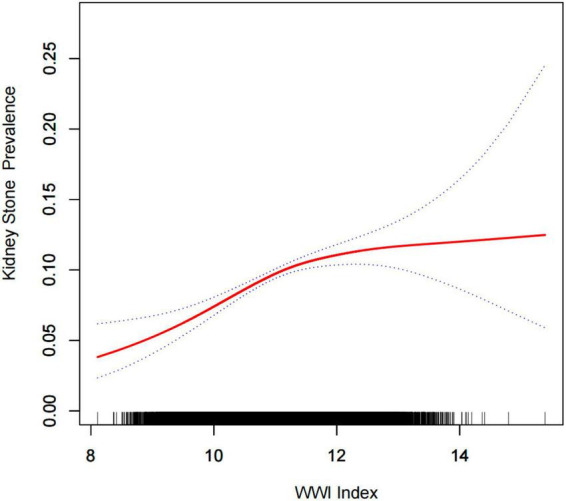
Density dose-response relationship between WWI index with kidney stone prevalence. The area between the upper and lower dashed lines is represented as 95% CI. Each point shows the magnitude of the WWI index and is connected to form a continuous line. Adjusted for all covariates except effect modifier.

Multiple regression analyses showed a positive association between WWI index and stone groups in both the crude and minimally/fully adjusted models with various adjustments for the effect of confounders on the association. In the fully adjusted model, each unit increase in the WWI index was associated with a 20% increased risk of kidney stones (OR = 1.20, 95%CI: 1.12, 1.28). In the sensitivity analysis, when the WWI index was changed to the third tertile, logistic regression showed a significant 41% increased risk of kidney stones in tertile 3 (OR = 1.41, 95%CI: 1.23, 1.61). Furthermore, when *WWI* was grouped by tertiles, we found that this positive association remained and became more pronounced with increasing WWI (P for trend < 0.01) (Table 2). Generalized additive model and smooth curve fitting were used to further explore the relationship between WWI index and kidney stones. Our results indicate a non-linear positive correlation between WWI index and kidney stones, with the saturation threshold effect analysis and the most important threshold value at 11.02 (Figure 2 and Table 3).

**TABLE 2:** Logistic regression analysis between WWI index with kidney stone prevalence.

Characteristic	Model 1 OR (95% CI)	Model 2 OR (95% CI)	Model 3 OR (95% CI)
WWI Index	1.52 (1.45, 1.59)	1.38 (1.31, 1.47)	1.20 (1.12, 1.28)
Categories			
Tertile 1	1	1	1
Tertile 2	1.69 (1.51, 1.88)	1.44 (1.29, 1.62)	1.28 (1.14, 1.44)
Tertile 3	2.37 (2.14, 2.62)	1.85 (1.65, 2.09)	1.41 (1.23, 1.61)
P for trend	<0.01	<0.01	<0.01

Model 1 was adjusted for no covariates; Model 2 was adjusted for age, gender, race, marital status and education; Model 3 was adjusted for covariates in Model 2 + diabetes, blood pressure, PIR, total water, total kcal, total sugar, total fat, smoked, physical activity, alcohol use, serum cholesterol, serum uric acid, coronary artery disease, serum glucose, serum calcium, serum triglycerides, serum creatinine and cancers were adjusted.

**TABLE 3:** Subgroup analysis between WWI index with kidney stone prevalence.

Characteristic	Model 1 OR (95% CI)	Model 2 OR (95% CI)	Model 3 OR (95% CI)
**Stratified by gender**			
Male	1.86 (1.73, 1.99)	1.34 (1.23, 1.47)	1.15 (1.03, 1.28)
Female	1.43 (1.34, 1.54)	1.37 (1.27, 1.48)	1.18 (1.08, 1.30) 0
**Stratified by race**			
Mexican American	1.43 (1.29, 1.58)	1.28 (1.13, 1.45)	1.21 (1.04, 1.39)
White	1.49 (1.39, 1.58)	1.41 (1.30, 1.53)	1.19 (1.08, 1.32)
Black	1.46 (1.29, 1.65)	1.31 (1.13, 1.51)	1.17 (0.98, 1.40)
Other race	1.75 (1.49, 2.06)	1.63 (1.34, 2.00)	1.31 (1.03, 1.67)
**Stratified by age (years)**			
20–39	1.41 (1.27, 1.57)	1.41 (1.26, 1.58)	1.32 (1.13, 1.54)
40–59	1.44 (1.32, 1.58)	1.54 (1.40, 1.69)	1.30 (1.15, 1.47)
60–85	1.19 (1.10, 1.29)	1.28 (1.17, 1.40)	1.12 (1.01, 1.24)
**Stratified by hypertension**			
Yes	1.24 (1.15, 1.33)	1.25 (1.14, 1.36)	1.16 (1.05, 1.28)
No	1.53 (1.43, 1.63)	1.40 (1.29, 1.51)	1.22 (1.11, 1.35)
**Stratified by diabetes**			
Yes	1.17 (1.04, 1.32)	1.21 (1.05, 1.39)	1.10 (0.94, 1.29)
No	1.48 (1.40, 1.56)	1.34 (1.26, 1.43)	1.22 (1.13, 1.32)

Model 1 = no covariates were adjusted.

Model 2 = Model 1 + age, gender, race, marital status and education were adjusted.

Model 3 = adjusted for all covariates except effect modifier.

### 3.3 Subgroup analysis

Subgroup analyses were performed to assess the robustness of the association between WWI index and kidney stones. Results (Table 4):

**TABLE 4:** Two-piecewise linear regression and logarithmic likelihood ratio test explained the threshold effect analysis of WWI index with kidney stone prevalence.

WWI index	ULR test OR (95% CI)	PLR test OR (95% CI)	LRT test *P*-value
<11.02	1.20 (1.12, 1.28)	1.48 (1.29, 1.70)	<0.0001
≥11.02		1.07 (0.98, 1.18)	

ULR, univariate linear regression; PLR, piecewise linear regression; LRT, logarithmic likelihood ratio test, statistically significant: *p* < 0.05.

• Male group (OR = 1.15, 95% CI:1.03, 1.28),
• Female group (OR = 1.18, 95% CI:1.08, 1.30),
• Age < 40 years group (OR = 1.32, 95% CI:1.13, 1.54),
• Age 40–59 years old group (OR = 1.30, 95% CI:1.15, 1.47),
• Age 60 years group (OR = 1.12, 95% CI:1.01, 1.24),
• The Mexican-American group (OR = 1.21, 95% CI:1.04, 1.39),
• White group (OR = 1.19, 95% CI:1.08, 1.32),
• Black group (OR = 1.17, 95% CI:1.00, 1.40),
• Other people group (OR = 1.31, 95% CI:1.03, 1.67),
• Hypertension group (OR = 1.16, 95% CI:1.05, 1.28),
• Non-hypertensive group (OR = 1.22, 95% CI:1.11, 1.35),
• Diabetes group (OR = 1.10, 95% CI:0.94, 1.29),
• Non-diabetic group (OR = 1.22, 95% CI:1.13, 1.32).

## 4 Discussion

In this nationally representative cross-sectional study based on the NHANES database, no epidemiologic study to date has reported an association between WWI index and kidney stone levels. Therefore, after adjusting for the association between WWI index and kidney stones with sociodemographic, laboratory, personal history, dietary, and comorbidity data, we found that a high WWI index was associated with an increased prevalence of kidney stones in the United States. Our study shows that each unit increase in the WWI index is associated with a 20% increased risk of kidney stones. To provide a more intuitive demonstration of the relationship between WWI index and kidney stone incidence, we used a generalized additive model and smooth curve fitting. The results showed a non-linear positive correlation between the WWI index and the incidence of kidney stones. These results provide strong evidence that obesity management as assessed by WWI can reduce the incidence of kidney stones.

Obesity and kidney stones are current public health concerns worldwide. Many previous studies have reported that obesity is associated with increased prevalence and recurrence of kidney stones (9, 26, 27). However, previous studies have focused on the relationship between the traditional obesity index, BMI, and kidney stones. A recent study found that small height was associated with stone formation but not weight (28), which may be due to the limitations of BMI (29) and the presence of the obesity paradox (22). In order to better explore the relationship between obesity and kidney stones, some recent studies tend to use non-traditional obesity indicators as a measure of obesity, such as visceral adiposity index (30), android-to-gynoid ratio (31), and so on. To the best of our knowledge, this is the first cross-sectional human study to evaluate the association between WWI and kidney stones. WWI partially attenuates the effect of BMI and better reflects true central obesity independent of body weight as a newly developed obesity index derived from the integration of waist circumference and body weight. However, increased visceral fat in central obesity may be more likely to reflect adverse metabolic characteristics, which is receiving increasing attention from researchers (12). Cardiovascular disease (19), proteinuria (20), and hyperuricemia (22) are just a few of the areas where WWI is being investigated. According to our results, it is confirmed that there is a very clear correlation between the WWI index and the prevalence of kidney stones. In order to find a specific population adapted to the WWI index that may better prevent the occurrence of kidney stones, we conducted a subgroup analysis. In the gender analysis, the effect of the WWI index on the prevalence of kidney stones was slightly higher in the female group than in the male group. At present, many studies have found that the difference between the risk of urolithiasis between men and women is gradually decreasing (32, 33), and our results are basically consistent with previous studies. In analyzing the age subgroups, we found that the correlation between high WWI index and the prevalence of urolithiasis was stronger in the younger age group compared to patients with high WWI index in the older group. This finding is encouraging, and if we can manage and control the WWI index at a young age, we can better prevent the occurrence of kidney stones. This result is similar to that reported by Hou et al. (30). In the gender group, the WWI index had the least effect on the prevalence of kidney stones, and the possible reason was that the black group seemed to be less susceptible to obesity than other ethnic groups (34). Finally, in the stratification of hypertension and diabetes, we found an interesting phenomenon, in non-hypertensive and non-diabetic population, WWI index and the prevalence of kidney stones have a stronger association, based on Zheng (35) and Shen (5), we believe that our results are reasonable. Further prospective cohort studies are needed to confirm causality.

The underlying mechanism of the association between obesity and kidney stones has not been completely clarified. Excess fat in obese patients’ livers affects purine metabolism, increasing uric acid production and uric acid excretion, leading to a higher frequency of uric-acid stones (20, 36). Adipocytes as endocrine tissue can secrete related adipokines through paracrine, autocrine and endocrine tissues, among which the most typical manifestation is hypoadiponectinemia (37), which may lead to increased production of reactive oxygen species (38, 39), leading to renal tubular epithelial cell injury, and renal cell injury and inflammation have been confirmed in the pathogenesis of idiopathic stone disease (40). At the same time, obesity can lead to insulin resistance, which can lead to excess uric acid. While insulin resistance increases citrate uptake in the renal tubules and reduces urinary citrate, which is a major risk factor for calcium stone formation (5). There is also the possibility that accumulation of fat in kidney tissue may trigger lipotoxic effects. Specifically, the accumulation of non-esterified fatty acids in renal cells can potentially disrupt cellular metabolism, leading to a reduction in ammonia production. This reduction in ammonia production may result in renal damage, thus promoting the formation of kidney stones (41). Additionally, increased fat content may be associated with elevated urate levels. The increase in urate is due to enhanced absorption of urate in the intestines and increased endogenous urate synthesis, which can also contribute to the formation of kidney stones (42).

Our study has the following strengths. NHANES strictly followed the well-designed study protocol, considering the sample weight problem, with good consistency, so the results are widely applicable to the general population in the United States, while our sample size is large enough to conduct relevant subgroup analysis to verify the robustness of the results. Inevitably, however, our study still has some shortcomings: (1) The study has a cross-sectional design, so we cannot determine the causal relationship between WWI and the prevalence of kidney stones. (2) Despite our adjustment for possible covariates, confounding due to unknown variables may remain. (3) Our kidney stone variables were obtained by questionnaire, and recall bias is inevitable; data on the exact type and volume of stones were not available, and some asymptomatic kidney stones may have adversely affected our study.

## 5 Summary

This study suggests that elevated WWI levels are associated with a higher prevalence likelihood of kidney stones and that obesity management, as assessed by WWI, may benefit kidney health, particularly in younger adults. However, further studies are needed to validate our findings.

## Data availability statement

The original contributions presented in this study are included in this article/supplementary material, further inquiries can be directed to the corresponding authors.

## Ethics statement

The NCHS Research Ethics Review Committee approved the NHANES survey protocol (https://www.cdc.gov/nchs/nhanes/irba98.htm) and all participants of the study provided informed written consent. The NHANES database is open to the public and therefore the ethical review of this study was exempt.

## Author contributions

ZG, LY, and YH: data analysis and manuscript writing. LY, YC, and MZ: study design and statistical advice. YC and JH: manuscript editing. YC, JH, and YH: validation and review. JH and YH: quality control. All authors agreed on the journal to which the article was to be submitted and agreed to take responsibility for all aspects of the work.

## Footnotes

1
www.empowerstats.com

2
http://www.R-project.org

## References

[1] Mao W Zhang L Sun S Wu J Zou X Zhang G Physical activity reduces the effect of high body mass index on kidney stones in diabetes participants From the 2007-2018 NHANES cycles: a cross-sectional study. *Front Public Health.* (2022) 10:936552. 10.3389/fpubh.2022.936552 35844866 PMC9283863

[2] Zhang G Zou X Mao W Chen M. Heterocyclic aromatic amines and risk of kidney stones: a cross-sectional study in US adults. *Front Public Health.* (2022) 10:935739. 10.3389/fpubh.2022.935739 35910865 PMC9330616

[3] Rule A Lieske J Li X Melton L III Krambeck A Bergstralh E. The ROKS nomogram for predicting a second symptomatic stone episode. *J Am Soc Nephrol.* (2014) 25:2878–86. 10.1681/ASN.2013091011 25104803 PMC4243346

[4] Ferraro P Curhan G D’Addessi A Gambaro G. Risk of recurrence of idiopathic calcium kidney stones: analysis of data from the literature. *J Nephrol.* (2017) 30:227–33. 10.1007/s40620-016-0283-8 26969574

[5] Shen X Chen Y Chen Y Liang H Li G Hao Z. Is the METS-IR index a potential new biomarker for kidney stone development. *Front Endocrinol.* (2022) 13:914812. 10.3389/fendo.2022.914812 35909543 PMC9329808

[6] Hyams E Matlaga B. Economic impact of urinary stones. *Transl Androl Urol.* (2014) 3:278–83. 10.3978/j.issn.2223-4683.2014.07.02 26816777 PMC4708578

[7] Alexander R Hemmelgarn B Wiebe N Bello A Morgan C Samuel S Kidney stones and kidney function loss: a cohort study. *BMJ.* (2012) 345:e5287. 10.1136/bmj.e5287 22936784 PMC3431443

[8] Taylor E Stampfer M Curhan G. Diabetes mellitus and the risk of nephrolithiasis. *Kidney Int.* (2005) 68:1230–5. 10.1111/j.1523-1755.2005.00516.x 16105055

[9] Taylor E Stampfer M Curhan G. Obesity, weight gain, and the risk of kidney stones. *JAMA.* (2005) 293:455–62. 10.1001/jama.293.4.455 15671430

[10] Holwerda S Gangwish M Luehrs R Nuckols V Thyfault J Miles J Concomitantly higher resting arterial blood pressure and transduction of sympathetic neural activity in human obesity without hypertension. *J Hypertens.* (2023) 41:326–35. 10.1097/HJH.0000000000003335 36583358 PMC9812452

[11] Dierkes J Dahl H Lervaag Welland N Sandnes K Sæle K Sekse I High rates of central obesity and sarcopenia in CKD irrespective of renal replacement therapy - an observational cross-sectional study. *BMC Nephrol.* (2018) 19:259. 10.1186/s12882-018-1055-6 30305034 PMC6180401

[12] Thomas E Frost G Taylor-Robinson S Bell J. Excess body fat in obese and normal-weight subjects. *Nutr Res Rev.* (2012) 25:150–61. 10.1017/S0954422412000054 22625426

[13] Koenen M Hill M Cohen P Sowers J. Obesity, adipose tissue and vascular dysfunction. *Circ Res.* (2021) 128:951–68. 10.1161/CIRCRESAHA.121.318093 33793327 PMC8026272

[14] Stefan N. Causes, consequences, and treatment of metabolically unhealthy fat distribution. *Lancet Diabetes Endocrinol.* (2020) 8:616–27. 10.1016/S2213-8587(20)30110-8 32559477

[15] Kielstein J Pontremoli R Burnier M. Management of hyperuricemia in patients with chronic kidney disease: a focus on renal protection. *Curr Hypertens Rep.* (2020) 22:102. 10.1007/s11906-020-01116-3 33128170 PMC7599161

[16] Andreoli A Garaci F Cafarelli F Guglielmi G. Body composition in clinical practice. *Eur J Radiol.* (2016) 85:1461–8. 10.1016/j.ejrad.2016.02.005 26971404

[17] Park Y Kim N Kwon T Kim SG. A novel adiposity index as an integrated predictor of cardiometabolic disease morbidity and mortality. *Sci Rep.* (2018) 8:16753. 10.1038/s41598-018-35073-4 30425288 PMC6233180

[18] Kim N Park Y Kim N Kim S. Weight-adjusted waist index reflects fat and muscle mass in the opposite direction in older adults. *Age Ageing.* (2021) 50:780–6. 10.1093/ageing/afaa208 33035293

[19] Li Q Qie R Qin P Zhang D Guo C Zhou Q Association of weight-adjusted-waist index with incident hypertension: the rural chinese cohort study. *Nutr Metab Cardiovasc Dis.* (2020) 30:1732–41. 10.1016/j.numecd.2020.05.033 32624344

[20] Qin Z Chang K Yang Q Yu Q Liao R Su B. The association between weight-adjusted-waist index and increased urinary albumin excretion in adults: a population-based study. *Front Nutr.* (2022) 9:941926. 10.3389/fnut.2022.941926 36034904 PMC9412203

[21] Ding C Shi Y Li J Li M Hu L Rao J Association of weight-adjusted-waist index with all-cause and cardiovascular mortality in China: a prospective cohort study. *Nutr Metab Cardiovasc Dis.* (2022) 32:1210–7. 10.1016/j.numecd.2022.01.033 35277327

[22] Zhao P Shi W Shi Y Xiong Y Ding C Song X Positive association between weight-adjusted-waist index and hyperuricemia in patients with hypertension: the China H-type hypertension registry study. *Front Endocrinol.* (2022) 13:1007557. 10.3389/fendo.2022.1007557 36277696 PMC9582276

[23] Yang J Li Y Liu Q Li L Feng A Wang T Brief introduction of medical database and data mining technology in big data era. *J Evid Based Med.* (2020) 13:57–69. 10.1111/jebm.12373 32086994 PMC7065247

[24] Wu W Li Y Feng A Li L Huang T Xu A Data mining in clinical big data: the frequently used databases, steps, and methodological models. *Mil Med Res.* (2021) 8:44. 10.1186/s40779-021-00338-z 34380547 PMC8356424

[25] von Elm E Altman D Egger M Pocock S Gøtzsche P Vandenbroucke J. The strengthening the reporting of observational studies in epidemiology (STROBE) statement: guidelines for reporting observational studies. *Bull World Health Organ.* (2007) 85:867–72. 10.2471/blt.07.045120 18038077 PMC2636253

[26] Sorensen M Chi T Shara N Wang H Hsi R Orchard T Activity, energy intake, obesity, and the risk of incident kidney stones in postmenopausal women: a report from the Women’s Health Initiative. *J Am Soc Nephrol.* (2014) 25:362–9. 10.1681/ASN.2013050548 24335976 PMC3904570

[27] Bonneau H Badetti GR Varette I. A case of benign mesothelioma of the testicle in an infant]. *J Urol Nephrol.* (1965) 71:662–6. 5844866

[28] Ganz M Alessandro C Jacobs M Miller D Diah J Desroches B Association of height and prevalence of kidney stones. *Cureus.* (2022) 14:e32919. 10.7759/cureus.32919 36699765 PMC9872204

[29] Burton J Gray L Webb D Davies M Khunti K Crasto W Association of anthropometric obesity measures with chronic kidney disease risk in a non-diabetic patient population. *Nephrol Dial Transplant.* (2012) 27:1860–6. 10.1093/ndt/gfr574 21965589

[30] Hou B Shen X He Q Chen Y Xu Y Chen M Is the visceral adiposity index a potential indicator for the risk of kidney stones. *Front Endocrinol.* (2022) 13:1065520. 10.3389/fendo.2022.1065520 36531468 PMC9751392

[31] Li G Liang H Hao Y Huang Q Shen X Chen Y Association between body fat distribution and kidney stones: evidence from a US population. *Front Endocrinol.* (2022) 13:1032323. 10.3389/fendo.2022.1032323 36277687 PMC9585195

[32] Pearle M Calhoun E Curhan G. Urologic diseases in America project: urolithiasis. *J Urol.* (2005) 173:848–57. 10.1097/01.ju.0000152082.14384.d7 15711292

[33] Scales C Jr. Curtis L Norris R Springhart W Sur R Schulman K Changing gender prevalence of stone disease. *J Urol.* (2007) 177:979–82. 10.1016/j.juro.2006.10.069 17296391

[34] Guerrero R Vega G Grundy S Browning J. Ethnic differences in hepatic steatosis: an insulin resistance paradox. *Hepatology.* (2009) 49:791–801. 10.1002/hep.22726 19105205 PMC2675577

[35] Qin Z Zhao J Geng J Chang K Liao R Su B. Higher triglyceride-glucose index is associated with increased likelihood of kidney stones. *Front Endocrinol.* (2021) 12:774567. 10.3389/fendo.2021.774567 34912299 PMC8667164

[36] Matsuura F Yamashita S Nakamura T Nishida M Nozaki S Funahashi T Effect of visceral fat accumulation on uric acid metabolism in male obese subjects: visceral fat obesity is linked more closely to overproduction of uric acid than subcutaneous fat obesity. *Metabolism.* (1998) 47:929–33. 10.1016/s0026-0495(98)90346-8 9711987

[37] Kim S Yi H Won K Lee J Kim H. Association between visceral adipose tissue metabolism and Alzheimer’s disease pathology. *Metabolites.* (2022) 12:258. 10.3390/metabo12030258 35323701 PMC8949138

[38] Sharma K Ramachandrarao S Qiu G Usui H Zhu Y Dunn S Adiponectin regulates albuminuria and podocyte function in mice. *J Clin Invest.* (2008) 118:1645–56. 10.1172/JCI32691 18431508 PMC2323186

[39] Sharma K. The link between obesity and albuminuria: adiponectin and podocyte dysfunction. *Kidney Int.* (2009) 76:145–8. 10.1038/ki.2009.137 19404275

[40] Khan S. Is oxidative stress, a link between nephrolithiasis and obesity, hypertension, diabetes, chronic kidney disease, metabolic syndrome. *Urol Res.* (2012) 40:95–112. 10.1007/s00240-011-0448-9 22213019 PMC5683185

[41] Nuttall F. Body mass index: obesity, BMI, and health: a critical review. *Nutr Today.* (2015) 50:117–28. 10.1097/NT.0000000000000092 27340299 PMC4890841

[42] Pigna F Sakhaee K Adams-Huet B Maalouf N. Body fat content and distribution and urinary risk factors for nephrolithiasis. *Clin J Am Soc Nephrol.* (2014) 9:159–65. 10.2215/CJN.06180613 24202136 PMC3878707

